# Targeted and redox-responsive drug delivery systems based on carbonic anhydrase IX-decorated mesoporous silica nanoparticles for cancer therapy

**DOI:** 10.1038/s41598-020-71071-1

**Published:** 2020-09-02

**Authors:** Minmin Chen, Jinxia Hu, Lujing Wang, Yanru Li, Chenghao Zhu, Chen Chen, Ming Shi, Zhicheng Ju, Xichuan Cao, Zhuoqi Zhang

**Affiliations:** 1 School of Materials and Physics, China University of Mining and Technology, Xuzhou, 221116 People’s Republic of China; 2 School of Chemical Engineering and Technology, China University of Mining and Technology, Xuzhou, 221116 People’s Republic of China; 3 Department of Cardiology, Affiliated Hospital of Xuzhou Medical University, Xuzhou, 221002 People’s Republic of China; 4 Cancer Institute, Xuzhou Medical University, Xuzhou, 221002 People’s Republic of China

**Keywords:** Cancer therapy, Biomedical materials, Drug delivery, Nanoparticles

## Abstract

In this work, we developed a new antibody-targeted and redox-responsive drug delivery system “MSNs-CAIX” by binding the anti-carbonic anhydrase IX antibody (A-CAIX Ab) on the surface of mesoporous silica nanoparticles (MSNs) via disulfide linkages. The design of the composite particles “MSNs-CAIX” involved the synthesis and surface functionalization with thiol groups, 2,2′-dipyridyl disulfide and CAIX antibody. In vitro, CAIX capping the doxorubicin hydrochloric (DOX)-loaded nanoparticles (DOX@MSNs-CAIX) exhibited effectively redox-responsive release in the presence of glutathione (GSH) owing to the cleavage of the disulfide bond. Compared with CAIX negative Mef cells (mouse embryo fibroblast), remarkably more DOX@MSNs-CAIX was internalized into CAIX positive 4T1 cells (mouse breast cancer cells) by receptor-mediation. Tumor targeting in vivo studies clearly demonstrated DOX@MSNs-CAIX accumulated in tumors and induced more tumor cells apoptosis in 4T1 tumor-bearing mice. With great potential, this drug delivery system is a promising candidate for targeted and redox-responsive cancer therapy.

## Introduction

Cancer is the second leading cause of death globally with a growing incidence and mortality rates year by year^1,2^. Long-term or large dose use of chemotherapy drugs could induce tumor cell apoptosis, while serious side effects occurred to normal tissues and organs due to the lack of specificity^3–5^. In recent decades, increasing scientists are devoted to exploring nanomaterials as drug delivery carriers to overcome the problems based on their superior advantages^6–8^. Encapsulation of the anti-cancer drugs in the nanomaterials can keep their stability, improve solubility and bioavailability, as well as alter biodistribution^9,10^. Moreover, nanoparticles loaded with drugs could successfully cross physiological barriers to target sites, ultimately, the local drug concentration was significantly improved by effectively releasing drug concentration, and the damage to normal tissues was obviously reduced^11,12^.

Since the first-generation drug delivery system (DDS) was reported in 1965^13^, a wide range of nanoparticles have been investigated, including liposome^14–16^, polymer nanoparticles^17,18^, magnetic nanoparticles^19,20^, carbon nanomaterials^21,22^, gold nanoparticles^23,24^ and mesoporous silica nanoparticles (MSNs)^25–28^. Among these nanoparticles, integrating the advantage of silica and porous nanostructure, MSNs have been considered to be the promising candidate as the drug carrier^29^. Silica, approved as a food additive by the Food and Drug Administration (FDA), are classified as “Generally Recognized as Safe”^30^. Additionally, MSNs possess tailorable channel structure, adjustable pore size, high specific surface area, easy functionalization, which endow them with unique advantages to deliver various therapeutic agents^31,32^.

Utilizing the distinctive superiority of abundant active hydroxyl groups on the surface, MSNs were incorporated with appropriate ligands to satisfy the various application requirements of cancer therapy^33,34^. It is known that passive targeting through enhanced permeability and retention (EPR) effect of MSNs faces problems of varied microvascular permeability and increased interstitial pressure^6^. To actively target the tumor sites and reduce damage to normal tissues, diverse biological recognition ligands were modified on the outer surface of MSNs to specifically recognize the receptors overexpressed on tumor cells^35^, including small molecules, monoclonal antibodies, aptamers, peptides and proteins^36,37^. Zhang et al.^38^ designed MSNs nanoplatform coupled with folic acid (FA), which possessed a high targeting performance to Hela and MDA-MB-231 cells because FA specifically binded to the folate-receptor sites on the surface of cells. Er et al.^39^ constructed cetuximab-targeted MSNs, significantly enhancing the therapy effect on pancreatic tumors due to cetuximab monoclonal antibody efficiently targeting the epidermal growth factor receptor (EGFR).

Additionally, to prevent drugs premature and release drugs on demand at specific sites, various stimuli-responsive DDSs based on MSNs were designed including pH, redox, enzyme, light, magnetic stimuli-responsive drug release^40–42^. Among these, redox stimuli-responsive caused more appealing attention considering the abundant reducing glutathione (GSH) in the cancer cells. The concentration of GSH in the normal cellular cytoplasm is as high as 10 mM, which is significantly higher than that of extracellular fluid of tissues (2 μM). Notably, the presence of GSH in tumor cytoplasm cells is fourfold higher than that of normal cells^43^. Since disulfide bonds could be cleaved by GSH in cancer cells, redox-driven capped MSNs linked with a disulfide linker will release drugs on-demand^44^.

CAIX, as a peculiar member of the membrane-associated Carbonic anhydrase (CA) family, was firstly identified in 1994^45^. In general, CAIX is poorly expressed in normal tissues and highly expressed in various solid tumors, including bladder, uterine cervix, kidneys, esophagus, lungs, head and breast carcinomas^46,47^. Antibody is one of the most widely used targeting moieties based on the affinity and specificity^44^. Anti-CAIX antibody (A-CAIX Ab), as a potential targeted agent, applied to MSNs has rarely been studied directly. Furthermore, CAIX was drafted on the surface of MSNs via redox-responsive disulfide linkages, which could efficiently trigger drug release by GSH.

**Scheme 1:**
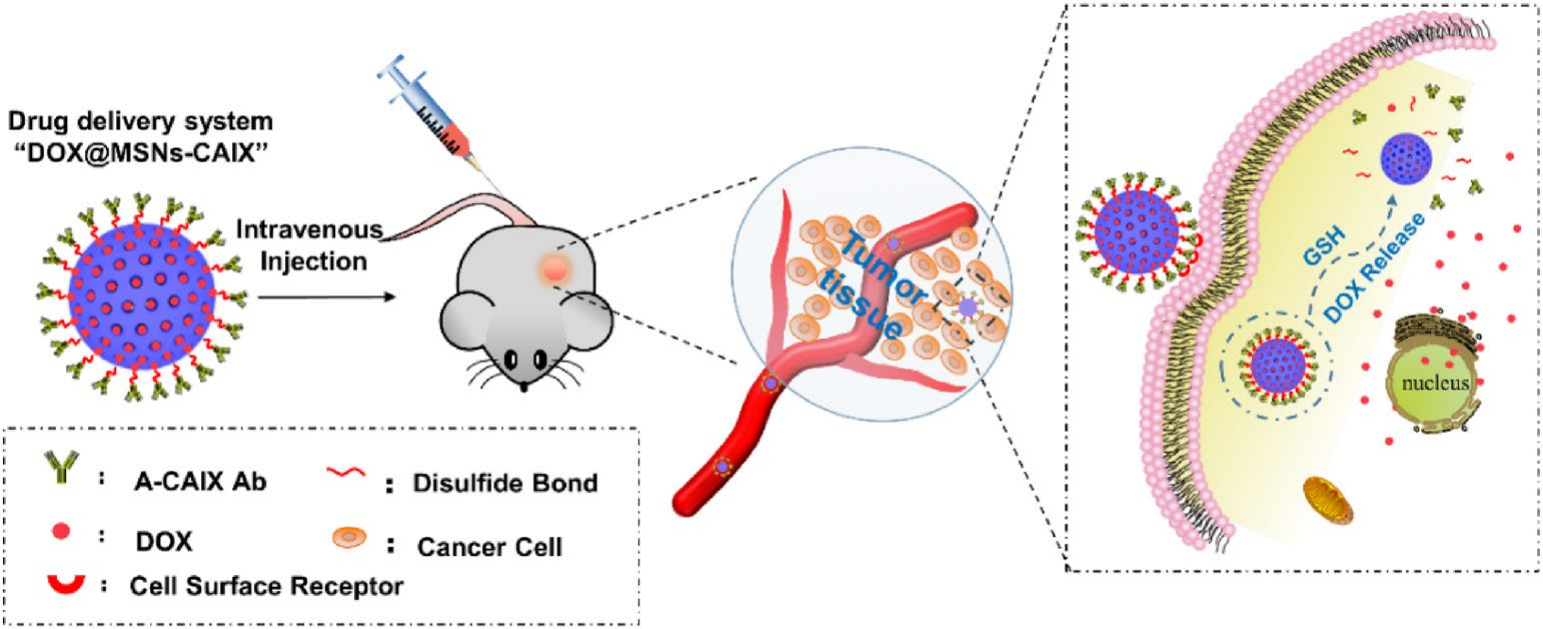
Illustration of A-CAIX Ab targeted mesoporous silica nanoparticles as a redox-responsive drug delivery system.

Here, we designed a novel targeted and redox-responsive drug delivery system “DOX@MSNs-CAIX” as shown in Scheme 1, in which MSNs were used as the vehicle to load chemotherapy drug doxorubicin (DOX) and CAIX grafted on MSNs by disulfide bonds. The drug loading capacity and the cytotoxicity of MSNs were investigated in vitro. Also, the endocytosis ability of MSNs-CAIX was investigated on CAIX expressed negative (Mef) and positive cells (4T1), respectively. DOX@MSNs-CAIX were injected to the mouse via tail vein in 4T1 tumor-bearing mice model to evaluate the targeting and the therapeutic effect. Results indicated that DOX@MSNs-CAIX could achieve GSH-triggered release and target to tumor sites. Thus, MSNs-CAIX are a promising drug delivery system for targeted cancer therapy.

## Methods

### Synthesis of MSNs, MSNs-SH, MSNs-S–S-P, MSNs-CAIX

MSNs were synthesized using the reported method^48^. 1.04 g, 25 wt% cetyltrimethyl ammonium chloride (CTAC) (Sigma) solution, 6.4 mL of deionized water, 0.02 g of diethanolamine (DEA), 0.9 g of ethanol were mixed and stirred in a water bath at 40 ℃ for 30 min. 0.73 mL of tetraethylorthosilicate (TEOS) (Sigma) was added dropwise into the mixture within 2 min followed by vigorously stirring for 2 h. The surfactant was removed by extraction at 80 ℃ ethanol acid solution (2 mL 37% HCl in 250 mL ethanol) for 8 h. Afterward, MSNs were washed thoroughly and dried under vacuum.

1 mL 3-mercaptopropyltrimethoxysilane (MPTMS) (Sigma) and 1 mL ethanol was mixed and stirred at room temperature for 24 h. Before the end of the reaction of MSNs, 200 μL of the mixture solution was added and stirred for another 2 h under nitrogen atmosphere. The mercaptopropyl-functionalized MSNs (MSNs-SH) were recovered by centrifugation, washed by ethanol three times. The surfactants were extracted as MSNs.

50 mg of MSNs-SH were dispersed in 10 mL phosphate buffer saline (PBS) of pH 4.6. 114.56 mg of 2,2′-dipyridyl disulfide (2,2′-dpd) was added and stirred for 24 h at room temperature. Particles were then centrifuged and washed with water. The 2,2′-dpd functionalized MSNs (MSNs-S-S-P) were obtained by vacuum freeze drying.

100 μg of A-CAIX Ab (clone No. MM0610-3B15, Abcam) was dissolved in 5 mL sodium borate buffer solution followed by 100 μL 2-Iminothiolane hydrochloride (2-IT) solution (0.8 mmol/L) and 200 μL 2.2 mol/L glycine sodium borate buffer solution added. The mixture was stirred for 1 h at room temperature. Sulfydryl-functionalized antibody protein (CAIX-SH) was purified by centrifuging using millipore ultrafiltration centrifuge tube (molecular interception is 30 KDa).

The as-prepared MSNs-S-S-P were suspended in 15 mL PBS (pH 7.4) containing 9.3 mL dimethyl sulfoxide (DMSO). Then, the CAIX-SH was added and stirred gently at room temperature for 24 h. Subsequently, the resulting particles were washed by water and collected by centrifuging. The antibody functionalized MSNs (MSNs-CAIX) were obtained and dried by freezing drier.

The amino-modified fluorescein isothiocyanate (FITC) (Alfa Aesar) was firstly prepared by adding 1 mg FITC and 5 µL 3-aminopropyltriethoxysilane (APTES) (Sigma) into 1 mL ethanol and stirred for 24 h away from light. Then, 20 µL amino-modified FITC was mixed with the prepared nanoparticles above (MSNs, MSNs-CAIX) for another 2 h by stirring in darkness at 40 ℃. FITC modified nanoparticles (MSNs-FITC and MSNs-CAIX-FITC) were collected by centrifugation and dried under vacuum.

### Characterization of the nanoparticles

The morphology, particle size and dispersion of MSNs were analyzed by HITACHI SU8220 field emission scanning electron microscope (SEM) with the working voltage at 10 kV and Tecnai G2 F20 (FEI, American) transmission electron microscopy (TEM) at an exciting voltage of 200 kV. The small angle X ray diffraction (XRD) pattern was recorded on D8 Advance (Bruker) by continuous scanning mode from 0.6° to 6°, with a scanning interval of 0.02°. The nitrogen (N_2_) adsorption desorption isotherm was operated on ASAP 2020 by static adsorption at 77 K. The analysis of infrared spectrum was revealed by Fourier Transform Infrared Spectroscopy (FTIR) Vertex 80v&HYPERION 2000 (Bruker, Germany) using KBr pellets as background. The raman spectrum was measured on Senterra Laser Confocal Raman Microspectroscopy (Bruker, Germany) using a charge coupled device (CCD) detector with an excitation wavelength of 785 nm and a cumulative number of 20 times. ^29^Si magic-angle-spinning nuclear-magnetic-resonance (^29^Si-MAS-NMR) was observed on Advance III HD 600MHZ (Bruker, Germany).

### Loading and redox-responsive release of DOX

For preparing DOX@MSNs-CAIX, doxorubicin hydrochloric (DOX) (Adams-beta) loaded MSNs-S-S-P (DOX@MSNs-S-S-P) were prior prepared. 1 mg/mL MSNs-S-S-P and 200 μg/mL DOX were suspended in PBS (pH 7.4). After ultrasonic dispersion, the mixture was shaken for 24 h at room temperature. After centrifugation, DOX@MSNs-S-S-P were collected by vacuum drying. DOX@MSNs were similarly prepared as DOX@MSNs-S-S-P. The supernatant was measured by ultraviolet–visible (UV–Vis) spectrometer (Evolution 60, Thermo) at 480 nm, the drug loading capacity and loading efficiency were calculated by the following formula:

$${\text{Loading Capacity }}({\text{mg/g}}) = \frac{{{\text{mass of DOX loaded in particles}}}}{{{\text{mass of particles}}}} \times 100\%$$

$${\text{Loading Efficiency }}(\% ) = \frac{{{\text{mass of DOX loaded in particles}}}}{{{\text{mass of DOX loaded particles}}}} \times 100\%$$

50 mg DOX@MSNs-S-S-P were dispersed in 15 mL PBS containing 9.3 mL DMSO following by adding the as-prepared CAIX-SH. The solution was stirred at room temperature for 24 h. After being washed with deionized water, DOX@MSNs-CAIX were collected by drying vacuum.

1 mg/mL DOX@MSNs-CAIX were added into PBS at different pH values (5.0, 6.0, 7.4) with or without GSH (0 mM, 2 mM, 5 mM, 10 mM). The resulting supernatant at different intervals (1 h, 3 h, 5 h, 7 h, 9 h, 12 h, 24 h and 48 h) was collected and measured by UV–Vis spectrometer. The cumulative drug release (%) was calculated as the following formula:

$${\text{Cumulative drug release }}(\% ) = \frac{{{\text{mass of DOX released from particles}}}}{{{\text{mass of DOX loaded in particles}}}} \times 100\%$$

### Cell culture

4T1-Luc (Luciferase) breast cancer cells were gifted from Jiangsu Center for the Collaboration and Innovation of Cancer Biotherapy. Mef cells (mouse embryo fibroblast cell line) were kindly gifted by Xuzhou Medical University. These two cell lines were cultured in Dulbecco’s modified Eagle medium (DMEM) containing 10% fetal bovine serum (FBS) and 1% penicillin–streptomycin in a humidified incubator at 37 °C and 5% CO_2_.

### In vitro cytotoxicity

Cell viability of 4T1 cells was investigated by Cell Counting Kit-8 (CCK-8) assay. Cells were seeded in 96 well plates (3 × 10^4^ cells per well). A certain concentration gradient solution (1 μg/mL, 5 μg/mL, 10 μg/mL, 25 μg/mL, 50 μg/mL, 100 μg/mL) of nanoparticles (MSNs, MSNs-SH, MSNs-S-S-P, MSNs-CAIX, DOX@MSNs, DOX@MSNs-CAIX) and equivalent concentrations of free DOX were dispersed in DMEM. After culturing overnight, cells were treated with the prepared particles solutions and cultured for 6, 12 and 24 h, respectively. Subsequently, cells were treated with CCK-8 agents (10 μL per well) and cultured for another 2 h. The absorbance value (i.e. OD) was measured by microplate spectrophotometer at a wavelength of 450 nm.

### Expression of CAIX in 4T1 and Mef cells

To confirm the expression of CAIX in 4T1 and Mef cells, quantitative real-time PCR (qRT-PCR) analysis was performed. Total RNA was extracted from 4T1 and Mef cells by trizol following the manufacturer’s instructions. For qRT-PCR, total RNA was extracted from 4T1 and Mef cells with trizol reagent (Invitrogen). 1 µg of RNA was converted to cDNA using the Revert Aidfirst Strand cDNA Synthesis Kit (Thermo) in a 20 μL reaction, and then 0.5 μL product was used in a 20 µL reaction mixture containing SYBR GreenER qPCR SuperMix Universal (Invitrogen) with CAIX primers: forward, 5′-GATTGAGGCTTCCTTCCC-3′ and reverse, 5′-TCTATCTTTGGTCCCACTTC-3′. The amplification cycle consisted of an initial step at 95 °C for 5 min, followed by 40 cycles of denaturation at 95 °C for 15 s and annealing at 60 °C for 1 min, and extension at 72 °C for 30 s. Samples were amplified independently at least three times. mRNA levels of CAIX were normalized to those of GAPDH, primer sequences as followed: forward, 5′-GCACAGTCAAGGCCGAGAAT-3′and reverse , 5′-GCCTTCTCCATGGTGGTGAA-3′.

### In vitro cellular uptake

Placing sterile microscope slides into 24-well plates, and 4T1 and Mef cells were seeded on the 24-well plates (3 × 10^4^ cells per well) for 24 h. Then the microscope slides with cells grown on their surface were transferred into one dish and co-cultured with DOX@MSNs, DOX@MSNs-CAIX and free DOX, respectively with the same DOX equivalent dose for 6 h. The cells were then fixed with paraformaldehyde (4%, g/mL) for 40 min followed by washing with PBS three times. Nuclei were stained by DAPI (blue) for 10 min and washed with PBS three times. The cellular uptake ability was examined by confocal laser scanning microscopy (CLSM, ZEISS LSM880) with the following channels: blue channel [2-(4-amidinophenyl)-6-indolecarbamidine dihydrochloride, DAPI] excited 405 nm, green channel (FITC) excited 488 nm and red channel (DOX) excited 561 nm.

### In vivo therapeutic assay

BALB/C mice (2 months old) were purchased from Ji Nan Peng Yue experimental animal breeding Co. Ltd., and the protocol was approved by the Institutional Animal Care and Use Committee of Xuzhou Medical University, according to National Institutes of Health guidelines. The study was approved by the Ethical Committee for Xuzhou Medical University. Mice were housed in clean plastic cages with a temperature of 25 ± 1 °C and humidity of 55–65% and maintained under specific pathogen-free conditions. The breast cancer model of the mice was established by subcutaneous inoculating 4T1-Luc breast cancer cells (5 × 10^6^ cells/mouse). When the tumor volume was approximately 300 mm^3^, the 4T1-Luc tumor-bearing mice were randomly divided into 3 groups (*n* = 3) and treated with PBS (control), DOX@MSNs, DOX@MSNs-CAIX, respectively. The samples in PBS at the equivalent dosage of 6 mg DOX per kg mice were injected via the tail vein at the 1st, 4th, 7th, 10th day, respectively. The body weights and the tumor volumes were recorded every two days, of which the volumes were calculated by the formula: V = W^2^ × L/2, (W and L: the width and the length of the tumor). After 11 days, the Luciferase-labeled Cancer Cells were detected by LB983 Night OWL II Small animal imaging system (Berthold Technologies).

### Statistical analysis

Statistical analysis was performed with prism 5 software. The two groups were compared using Student’s *t* test, for multiple-group comparisons, one-way ANOVA was used, followed by post hoc tests of Bonferroni or Fisher least significant difference as necessary. All data were expressed as mean ± standard deviation (SD) in triplicates. The data were considered to be significant when *P* < 0.05.

## Results and discussion

### Synthesis and characterization of CAIX targeted drug delivery system “MSNs-CAIX”

**Scheme 2:**
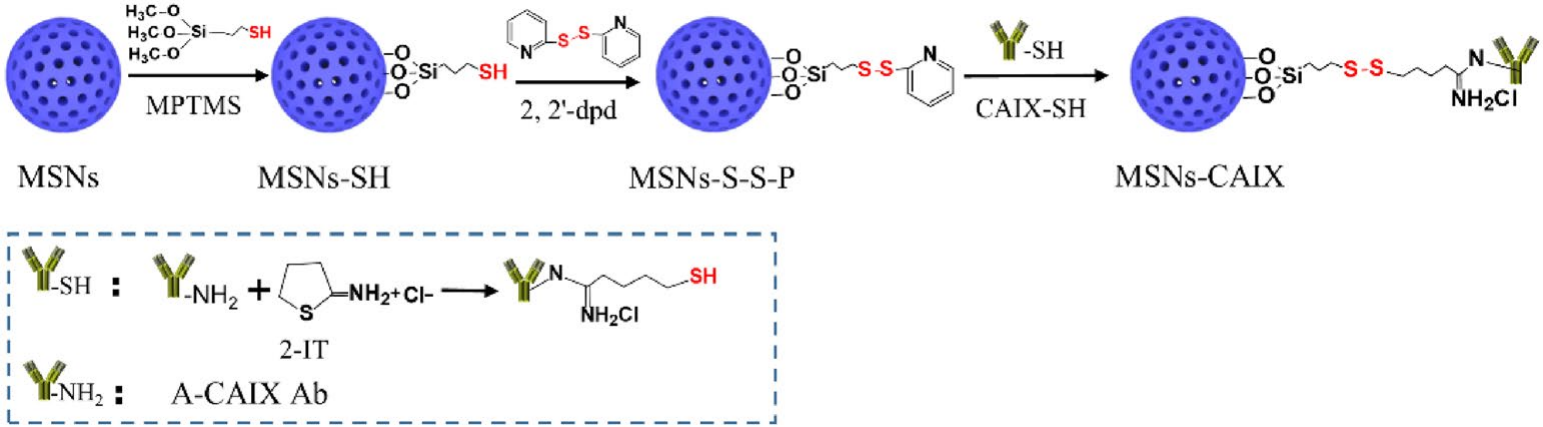
Synthetic route of CAIX targeted mesoporous silica nanoparticles “MSNs-CAIX”.

The detailed synthetic steps of A-CAIX Ab decorated MSNs (MSNs-CAIX) are depicted in Scheme 2. The MSNs were firstly prepared via a base-catalyzed sol–gel method with TEOS as the silica precursor, CTAC surfactant as the template and DEA as the catalyst. MSNs-SH were prepared by grafting MPTMS on the outer surface of MSNs. Subsequently, MSNs-S-S-P were synthesized by introducing 2,2′-dpd. A-CAIX Ab, as the targeting agent to enhance the tumor-targeted accumulation, was firstly modified with sulfhydryl group by introducing 2-IT to react with primary amine of CAIX. Finally, the sulfydryl-activated CAIX (CAIX-SH) was decorated on the MSNs-S-S-P surface (MSNs-CAIX) by disulfide linkage. The physicochemical properties of the prepared particles were comprehensively evaluated with transmission electron microscopy (TEM), scanning electron microscopy (SEM), small angle X ray diffraction (XRD), N_2_ isothermal adsorption, ^29^Si magic-angle-spinning nuclear-magnetic-resonance (^29^Si-MAS-NMR), fourier transform infrared spectroscopy (FTIR), raman spectroscopy.

**Figure 1:**
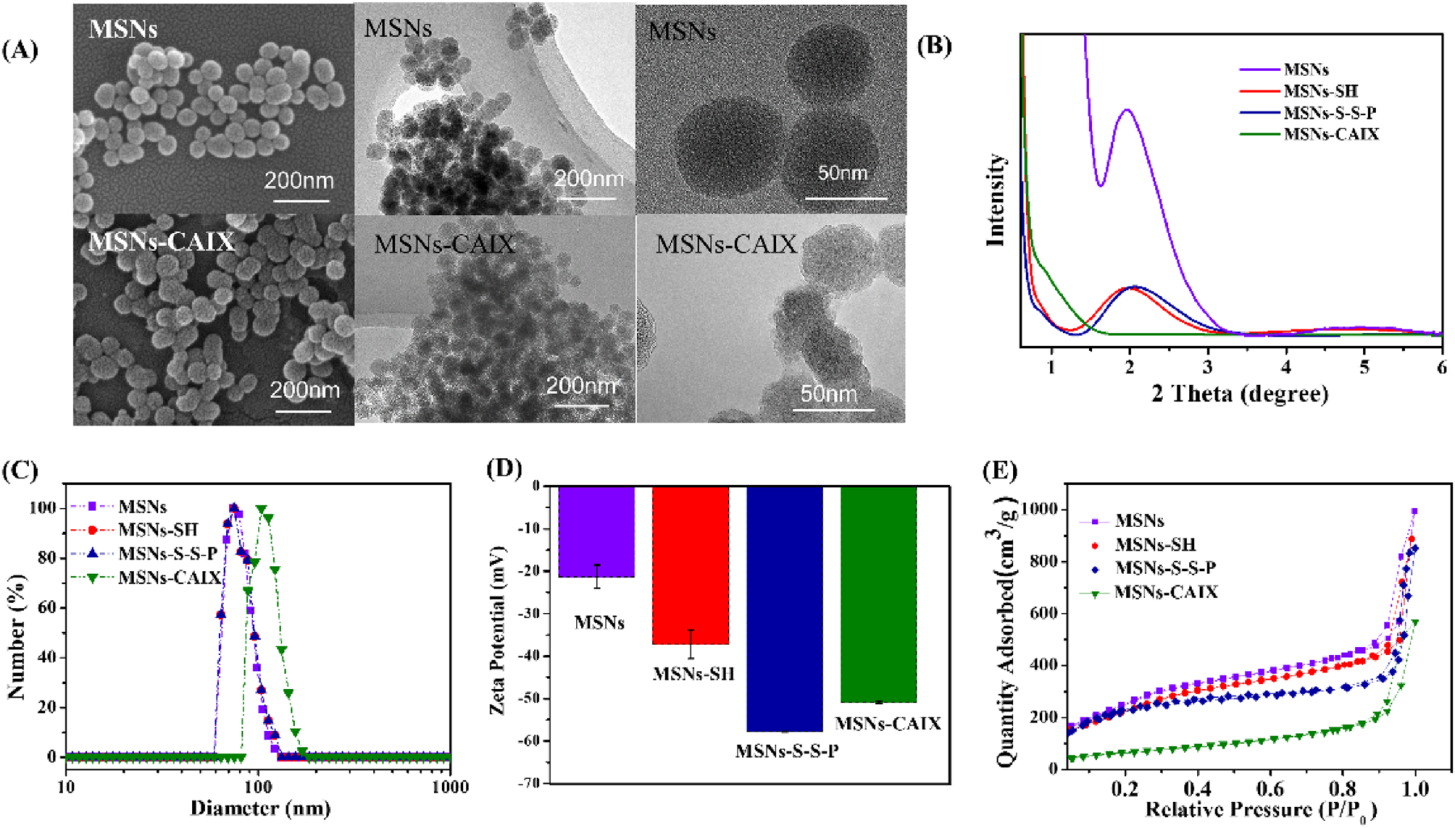
(**A**) SEM and TEM images of MSNs and MSNs-CAIX. (**B**) XRD patterns, (**C**) size distribution, (**D**) zeta potential, (**E**) nitrogen adsorption desorption isotherms of MSNs, MSNs-SH, MSNs-S-S-P and MSNs-CAIX.

The morphology and the sizes of the particles were revealed by SEM and TEM (Fig. 1A) images. The average diameter of these prepared particles was about 60 nm. The small angle powder X-ray diffraction patterns (XRD) of MSNs exhibited the diffraction peak (100) within the 2θ of 2°, the ordered mesoporous structure of MSNs-SH and MSNs-S-S-P was a little weaken after modification as shown in Fig. 1B. After capping with CAIX, the XRD pattern of MSNs-CAIX performed no peaks, indicating the pores of MSNs were capped by CAIX, which was also verified by the blurred pore structure from TEM images of MSNs-CAIX (Fig. 1A). The average hydrodynamic diameter of the prepared particles was presented in Fig. 1C, the diameter for MSNs, MSNs-SH and MSNs-S-S-P were approximately 73 nm. The size of MSNs-CAIX was 104 nm, larger than that displayed in TEM images, attributing to the slight aggregation after decorated CAIX. The zeta potential of the nanoparticles was shown in Fig. 1D, the zeta potential values of MSNs, MSNs-SH, MSNs-S-S-P and MSNs-CAIX were − 21 ± 2.78 mV, − 35 ± 3.35 mV, − 58 ± 0.12 mV, − 51 ± 0.27 mV, respectively. The changes were attributed to the different functional groups grafted on the surface of MSNs. The N_2_ adsorption–desorption curves of nanoparticles were shown in Fig. 1E. The specific surface area of bare MSNs was calculated to be 995 m^2^/g. Due to the modification of chemical functional groups, the specific surface area of MSNs-SH and MSNs-S-S-P slightly reduced to 881 m^2^/g and 833 m^2^/g, respectively, which still keep large surface area to load drugs. Moreover, after being wrapped with CAIX, the specific surface area of MSNs-CAIX significantly decreased to 239 m^2^/g, suggesting that CAIX was successfully linked on the surface of MSNs and sealed mesopore channels.

**Figure 2:**
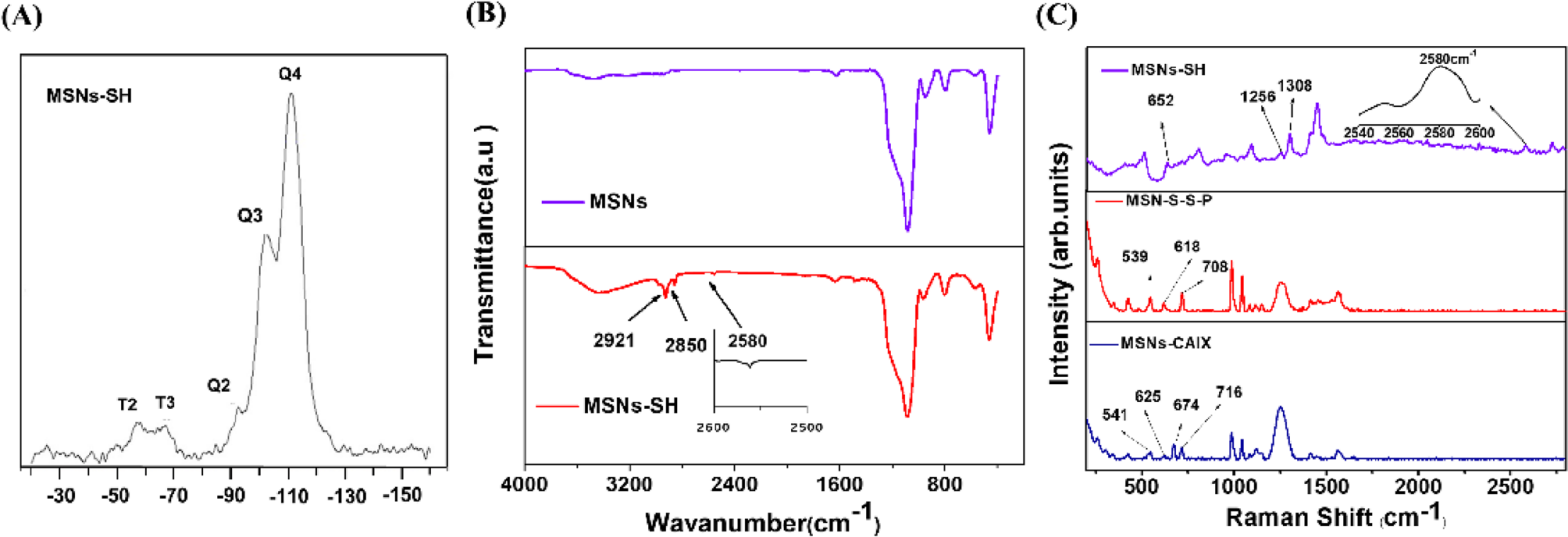
(**A**) ^29^Si-MAS-NMR of MSNs-SH. (**B**) The FT-IR of MSNs and MSNs-SH. (**C**) The Raman patterns of MSNs-SH, MSNs-S-S-P and MSNs-CAIX.

Both Q and T signals of MSNs-SH were found in the ^29^Si-MAS-NMR spectrum (Fig. 2A). The Q peaks were located at − 92 ppm (Q_2_), − 102 ppm (Q_3_), and − 111 ppm (Q_4_), exhibiting the typical Q_2-4_(Q_n_ = (SiO)_n_ (OH)_4-n_) network of the siloxane. The resonance peaks of T_2_ and T_3_ (T_2_ = (SiO)_2_ Si(OH) SH, T_3_ = (SiO)_3_SiSH) at − 57 ppm and − 67 ppm revealed that the sulfhydryl group was covalently bonded in the silica network successfully. FTIR spectra of MSNs and MSNs-SH was illustrated in Fig. 2B. Compared with MSNs, the vibration of C–H bond at 2,850 cm^−1^ and 2,921 cm^−1^ in MSNs-SH, was attributed to the successfully modified propylidene group. In addition, the characteristic peak 2,580 cm^−1^ can be identified as the SH-stretching vibration. The A-CAIX Ab was grafted and characterized by raman spectroscopy (Fig. 2C). In Fig. 2C, for MSNs-SH, the stretching vibration of SH at 2,580 cm^−1^ can be observed. Meanwhile, the oscillation of CH_2_ in the propyl-bridge between the silicon substrate and the sulfydryl group, namely CH_2_–Si and CH_2_–S, can be observed at 1,308 cm^−1^ and 1,256 cm^−1^, respectively. To sum up, sulfydryl group was successfully grafted onto the silica, which was consistent with FT-IR. For MSNs-S-S-P, three peaks can be clearly observed. They were disulfide bonds located at 539 cm^−1^, stretching vibration peaks of carbon–sulfur bonds at 618 cm^−1^ and 708 cm^−1^, indicating the successful introduction of disulfide bonds into the particles. For MSNs-CAIX, four peaks were observed. The disulfide peak moved to 541 cm^−1^ due to the change of the chemical environment. The stretching peak of the carbon–sulfur bond is located at 625 cm^−1^ and 716 cm^−1^. The new peak at 674 cm^−1^ is the vibration pattern of the carbon–sulfur bond corresponding to the propyl group of CAIX itself. The mentioned peaks showed that the antibody protein CAIX was successfully introduced into the surface of MSNs.

### Loading and redox-responsive release of DOX

**Figure 3:**
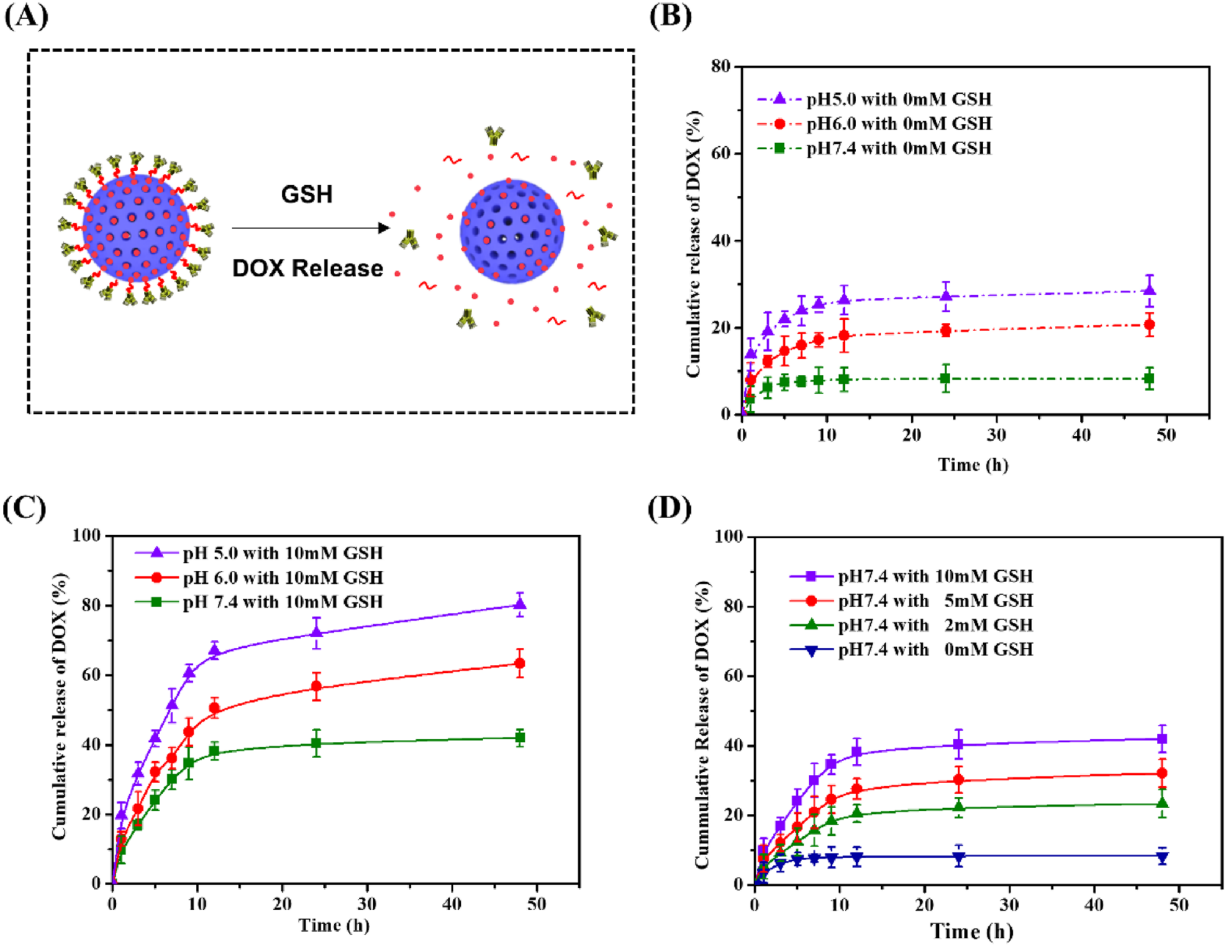
(**A**) Illustration of GSH-triggered DOX release from DOX@MSNs-CAIX. DOX release from DOX@MSNs-CAIX in PBS with (**B**) 0 mM GSH and (**C**) 10 mM GSH at different pH values (5.0, 6.0, 7.4). (**D**) DOX release from DOX@MSNs-CAIX in PBS at pH 7.4 with different concentrations of GSH (0 mM, 2 mM, 5 mM, 10 mM).

For DOX loading, the loading capacity and efficiency were 163 mg/g and 14%, respectively. GSH triggered redox-responsive DOX release behavior was investigated (Fig. 3A). In the absence of GSH, DOX cumulative release amount was 28%, 21% and 8%, respectively at pH 5.0, 6.0, 7.4 for 48 h (Fig. 3B). After adding 10 mM GSH into PBS at pH 5.0, 6.0, 7.4 (Fig. 3C), the cumulative release increased to 80%, 63% and 42%. The presence of GSH contributed to the cleavage of disulfide bonds between A-CAIX Ab and MSNs. Along with more A-CAIX Ab detached from MSNs, more DOX was released from the pores of MSNs. To furtherly verified the effect of GSH on redox-responsive release, the release properties of DOX@MSNs-CAIX was investigated in PBS at pH 7.4 with various concentrations of GSH (0 mM, 2 mM, 5 mM, 10 mM) (Fig. 3D). It was obvious that a higher concentration of GSH boosted more DOX release, indicating that DOX release behavior from DOX@MSNs-CAIX was GSH stimulation dependent. As known, the level of GSH in the cytoplasm of cancer cells is much higher than that of normal cells^44^, which would enhance the release due to the break of disulfide bonds on DOX@MSNs-CAIX. In addition, we can see that the release was pH-dependent, the lower pH, the higher cumulative release amount. The pH value of extracellular tumor tissues (6.5–6.8) tends to be more acidic than that of the normal tissues (7.4) and further decreases to 4.5–5 in lysosomes, 5.5–6.0 in endosomes^49^. The increased release in acidic environment facilitates pharmacotherapy aiming at tumor tissues.

### In vitro cytotoxicity

**Figure 4:**
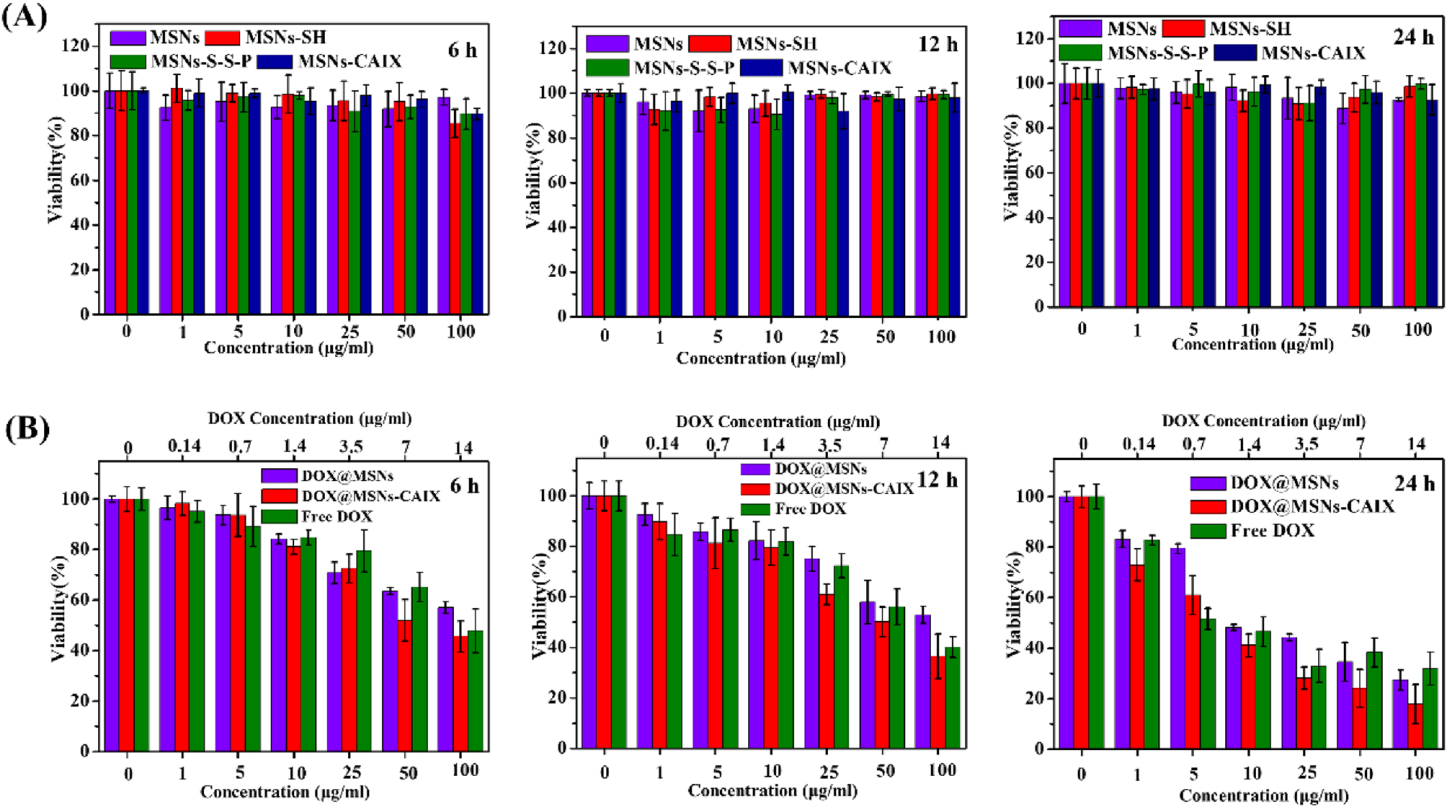
In vitro cytotoxicity of (**A**) MSNs, MSNs-SH, MSNs-S-S-P, MSNs-CAIX and (**B**) DOX@MSNs, DOX@MSNs-CAIX, free DOX cultured with 4T1 cells for 6 h, 12 h, 24 h, respectively.

The cytotoxicity of particles (MSNs, MSNs-SH, MSNs-S-S-P, MSNs-CAIX, DOX@MSNs-CAIX and free DOX) was performed via CCK-8 assay after incubation with 4T1 cells for 6, 12 and 24 h. As shown in Fig. 4A, all the nanoparticles exhibited low cytotoxicity against 4T1 cells over time at concentrations up to 100 μg/mL, indicating the particles were of great biocompatibility and the decorated group on MSNs surface did not bring extra cytotoxicity. To evaluate the cytotoxicity of the drug-loaded particles, a uniform solution of drug-loaded particles (DOX@MSNs, DOX@MSNs-CAIX) and free DOX molecules with the same drug concentration were prepared and cultured with 4T1 cells for 6, 12 and 24 h. As shown in Fig. 4B, DOX@MSNs, DOX@MSNs-CAIX and free DOX all inhibited 4T1 cells growth, and the cell viability declined with the increased concentration and the prolonging of culture time. Among them, DOX@MSNs-CAIX showed a significantly enhanced cytotoxicity. The inhibiting concentration 50% (IC_50_) values for 24 h of DOX@MSNs, DOX@MSNs-CAIX and DOX were calculated to be 2.5, 1.1 and 1.6 μg/mL of equivalent DOX concentration. DOX@MSNs-CAIX had the best in vitro inhibitory effects on 4T1 cells at the equal DOX concentration, attributing to the targeted A-CAIX Ab for specific binding the cells and easier cell internalization.

### CAIX expression and cellular uptake in 4T1 and Mef cells

**Figure 5:**
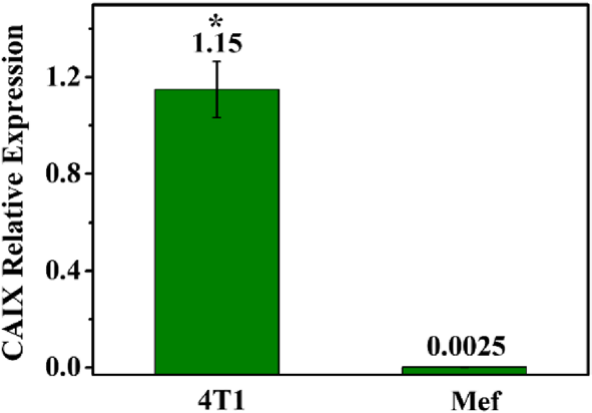
CAIX relative expression in 4T1 and Mef cells. (*P < 0.05 as compared with Mef group).

To assess CAIX expression in tumor cells (4T1) and normal cells (Mef), total CAIX RNA from 4T1 and Mef cells were extracted and detected by qRT-PCR technology with CAIX specific primers. As shown in Fig. 5, the level of CAIX was significantly higher expressed in 4T1 cell line, but almost undetected in Mef cell line.

**Figure 6:**
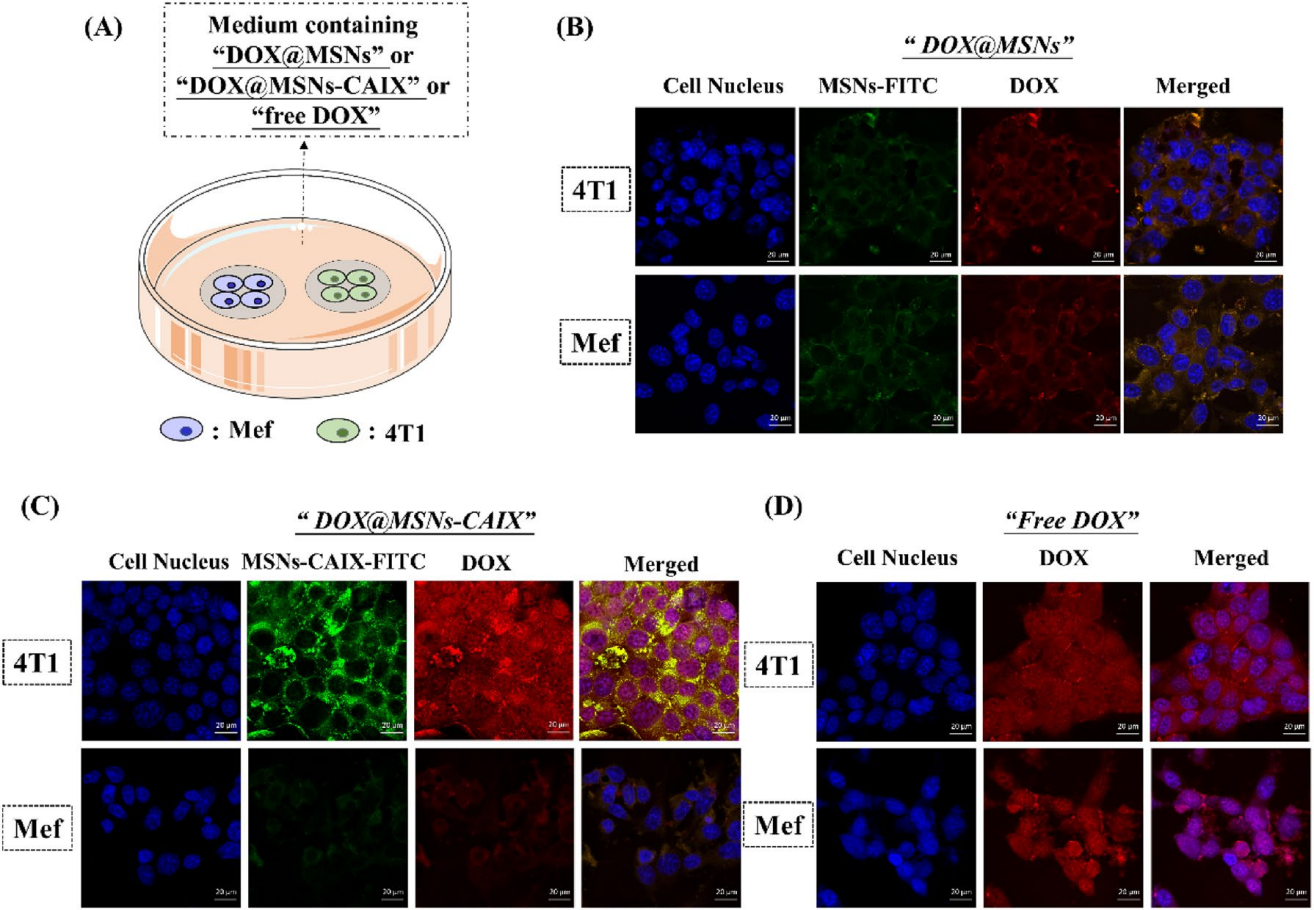
(**A**) Illustration of co-culture of 4T1 and Mef cells with DOX@MSNs, DOX@MSNs-CAIX and free DOX, respectively. CLSM images of 4T1 and Mef cells cultured with (**B**) DOX@MSNs, (**C**) DOX@ MSNs-CAIX, (**D**) Free DOX. Blue: cell nucleus staining by DAPI. Green: MSNs-CAIX labeled by FITC. Red: DOX.

In order to validate the targeted capacity of A-CAIX Ab, the CAIX expressed positive and negative cells (4T1 and Mef) grown on the microscope slides were transferred into one dish (Fig. 6A) and co-cultured with DOX@MSNs, DOX@MSNs-CAIX and free DOX, respectively with equivalent drug dose. As shown in Fig. 6B, for DOX@MSNs, MSNs-FITC (green) and DOX (red) were distributed in cytoplasm of 4T1 and Mef cells. On the contrary, for DOX@MSNs-CAIX, increasing fluorescent signal of MSNs-CAIX-FITC and DOX in 4T1 cells was found compared with Mef cells (Fig. 6C). And the released DOX was evenly distributed in cytoplasm and nucleus of 4T1, which was similar as the endocytosis behavior of free DOX by 4T1 (Fig. 6D). For CAIX positive 4T1 cells, A-CAIX Ab specifically recognized the CAIX antigen overexpressed on 4T1, effectively enhancing the internalization of drug-loaded particles. 4T1 and Mef cells were cultured in the same environment, with the same drug dose, MSNs-CAIX significantly delivery and release more DOX into CAIX positive 4T1 cells, implying the targeted capacity of A-CAIX Ab.

### In vivo therapeutic efficacy

**Figure 7:**
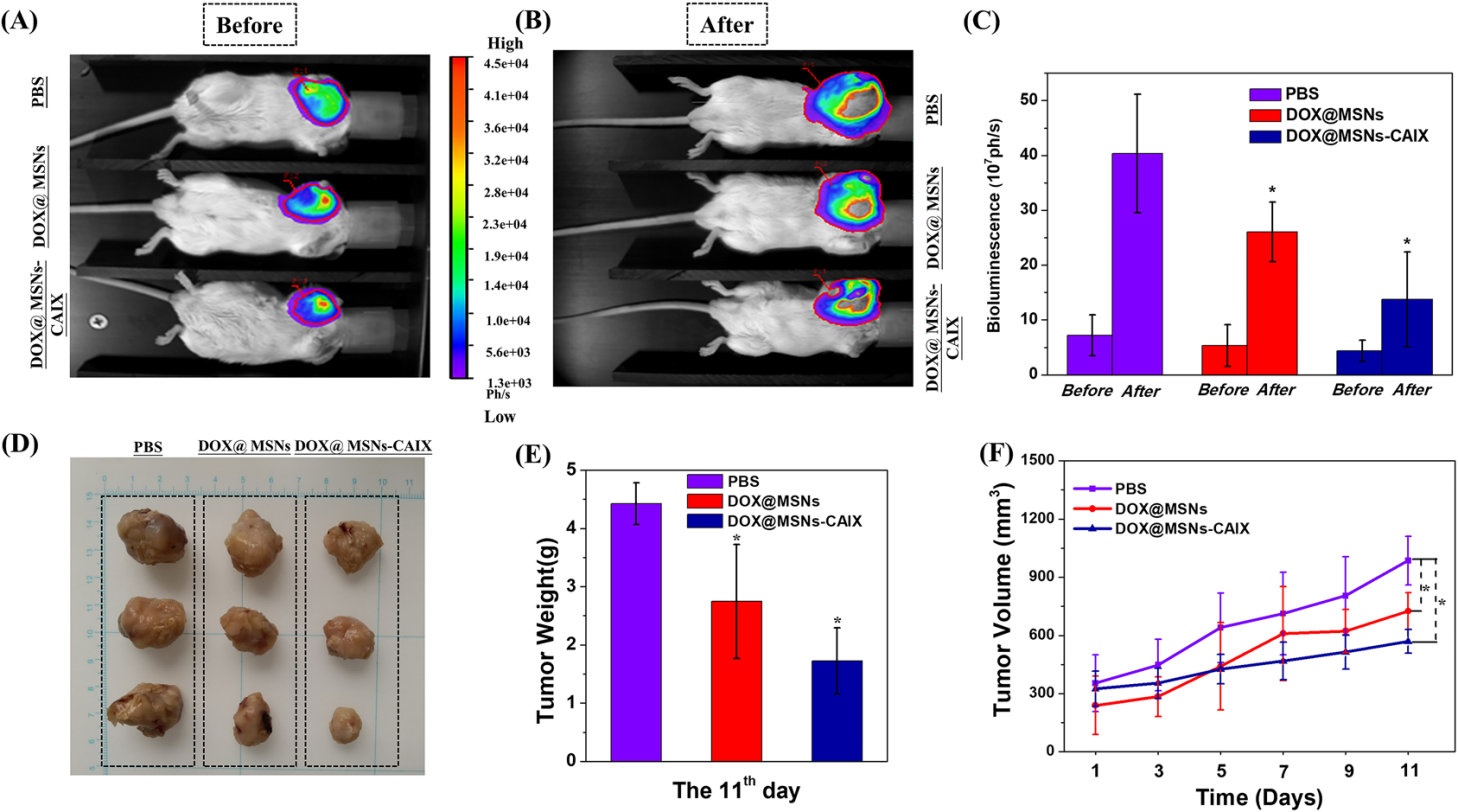
4T1-Luc tumor-bearing mice treated with different samples (PBS, DOX@MSNs and DOX@MSNs-CAIX) for 11 days. The tumors bioluminescence imaging before (**A**) and after (**B**) intervention with the above samples. (**C**) The tumors bioluminescence intensity before (the 0th day) and after (the 11th day) intervention with the above samples. (**D**)The tumors' photographs from the scarified mice. (**E**)The final average tumor weight. (**F**) The variation curves of average tumor volume. (*P < 0.05 as compared with PBS group).

To validate the in vivo targeted anti-cancer efficacy of “MSNs-CAIX”, 4T1-Luc tumor-bearing mouse model was established. The mice were treated with PBS, DOX@MSNs and DOX@MSNs-CAIX, respectively via tail intravenous injection for 11 days (Fig. 7A,B). Before receiving treatment, the tumor size of tumor-bearing mice in the three groups was little difference (Fig. 7A). After intervention for 11 days the tumors of the three groups all grew with time (Fig. 7B), however, the tumors in DOX@MSNs-CAIX group grew significantly slower than the other two groups due to its remarkably efficient tumor inhibition, and the corresponding bioluminescence intensity was quantitatively demonstrated in Fig. 7C. Then, the solid tumor tissues were removed, as shown in Fig. 7D,E, the size and the weight of tumors in DOX@MSNs-CAIX group were the smallest among these three groups. Also, the tumor growth rate of DOX@MSNs-CAIX group was significantly lower than the other two groups (Fig. 7F). These results demonstrated that DOX@MSNs-CAIX exhibited the most effective cancer therapeutic efficacy, which was mainly due to the precise delivery of the drug at the targeted tumor site.

**Figure 8:**
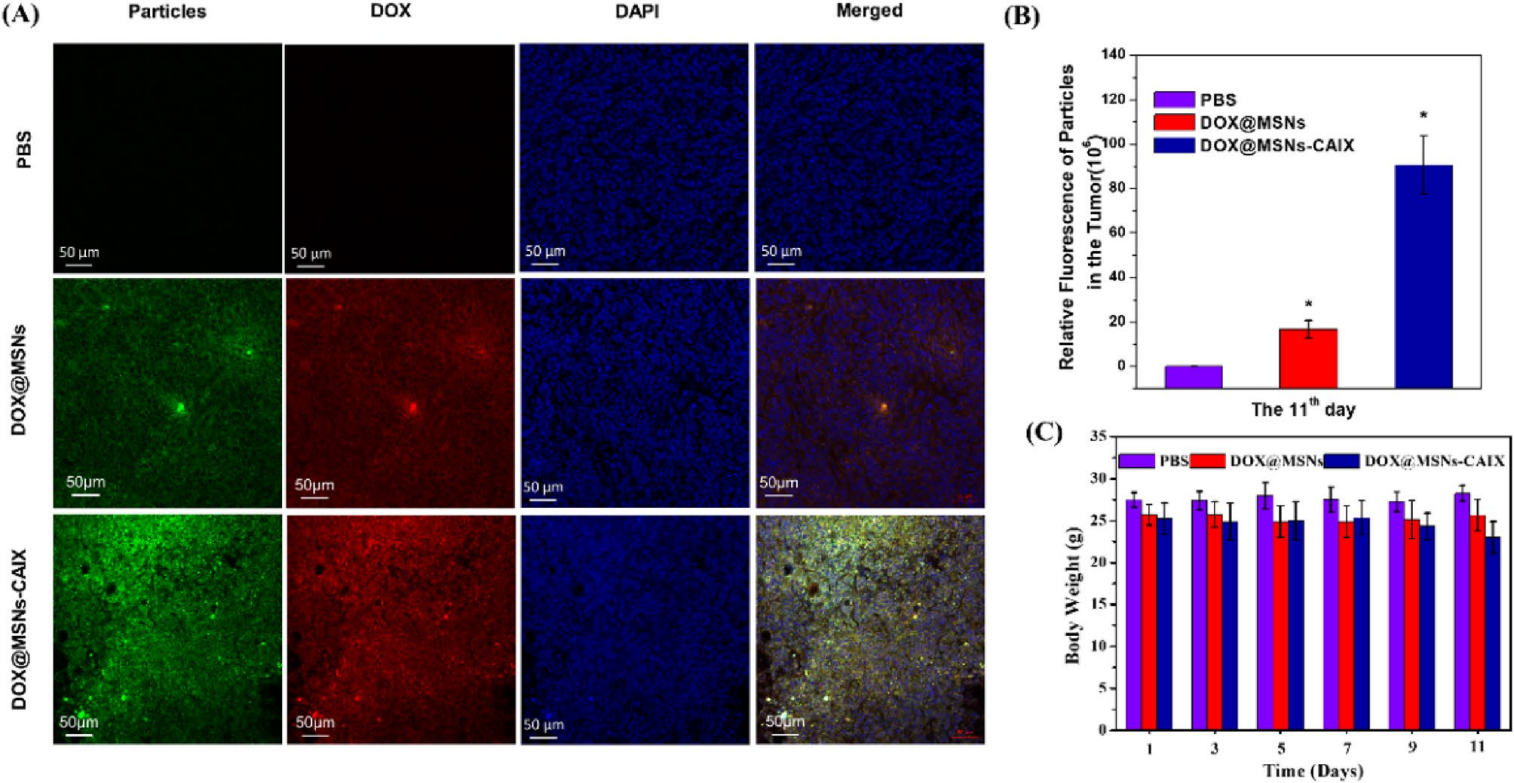
(**A**) Particles and DOX distribution in tumors after being treated with the above samples for 11 days (green: particles labeled by FITC; red: DOX; blue: cell nucleus staining by DAPI). (**B**) Particles relative fluorescence intensity in the tumor. (**C**) The variation of average body weight with time (*P < 0.05 as compared with PBS group).

The results were furtherly confirmed by the in vivo distribution of particles and DOX in the tumor tissues (Fig. 8A). It can be seen that the fluorescence signals of DOX (red) and MSNs-CAIX (green) in DOX@MSNs-CAIX group were stronger than those in DOX@MSNs group and PBS group. Furthermore, the quantitative analysis of the particles fluorescence intensity (Fig. 8B) showed that there were more DOX@MSNs-CAIX in the tumor due to the targeted capacity. Also as shown in Fig. 8C, negligible change in mouse body weight was measured in the experimental process, indicating that the MSNs and MSNs-CAIX have excellent biocompatibility, which could act as the promising drug delivery vehicle in vivo.

All the above results indicated that the A-CAIX Ab conjugated MSNs could significantly improve the anti-tumor therapy efficacy as the result of the fact that the targeted A-CAIX Ab could “navigate” the DOX loaded MSNs to the destination by specifical receptor mediated endocytosis, and the enriched drug cause more tumor cells apoptosis.

### Conclusions

In this study, we found that MSNs capped with A-CAIX Ab “MSNs-CAIX” could be used as a redox-responsive controlled-release and targeted delivery carrier. The model drug DOX could be released from the delivery system with GSH as a trigger. In vitro*,* the MSNs-CAIX could significantly facilitate cell internalization in CAIX-positive cancer cells owing to the targeted capacity of A-CAIX Ab. Moreover, in vivo*,* the DOX@MSNs-CAIX could suppress tumor growth more efficiently, which could act as the specific drug delivery vehicle for cancer therapy. Selecting MSNs as the vehicle with the versatile features, A-CAIX Ab as the targeted agent with specific recognition of the tumor cell surface, this exciting active targeted system MSNs-CAIX was worth being further developed for tumor targeting therapy.
